# Learning Lessons for Future Preparedness: Exploring Work Well-Being-Related Leadership Challenges among Paramedics during the Early Stage of the COVID-19 Pandemic—A Qualitative Study

**DOI:** 10.3390/nursrep13040142

**Published:** 2023-12-12

**Authors:** Jukka Pelli, Hilla Nordquist

**Affiliations:** 1 Faculty of Information Technology and Communication Sciences, Tampere University, Kalevantie 4, 33100 Tampere, Finland; 2 Department of Health Care and Emergency Care, South-Eastern Finland University of Applied Sciences, Pääskysentie 1, 48220 Kotka, Finland

**Keywords:** paramedic, emergency medical service, leadership, management, work well-being, emergency conditions, COVID-19, pandemic

## Abstract

The beginning of the COVID-19 pandemic majorly impacted the population and public services. In Finland, a state of emergency was declared to ensure the security of healthcare resources, and prehospital emergency medical service (EMS) organizations faced emergency conditions for the first time. This study explores the leadership challenges related to well-being experienced during the early phase of the pandemic. This qualitative study utilized reflective essay material written between August and November 2020 by experienced advanced-level paramedics (*n* = 30) who participated actively in EMS fieldwork at the beginning of the pandemic. The material (32,621 words) was analyzed with inductive content analysis. The work well-being-related leadership challenges experienced by paramedics during the early phase of the pandemic were divided into four upper categories: inadequate guidance, workplace reorganization, atmosphere mismanagement, and insufficient resources to cope. These upper categories were comprised of 17 subcategories. Several actions can be taken to enhance personnel well-being and prepare for similar challenges. Guidance and support should be clear and timely. Visible leadership should be emphasized and enhanced with modern communication. Efforts should be made to strengthen the work atmosphere to support those on the front lines of healthcare. This study was not registered on a publicly accessible registry.

## 1. Introduction

Effective leadership is an integral component of fostering an organization to achieve its goals. Leadership sets the tone for the entire workforce, providing vision, direction, motivation, and work well-being. Its effects on work well-being become even more important during times of crisis, where emotional and physical stress among employees can be severely increased, and building employee resiliency is needed [1].

The COVID-19 pandemic has impacted local emergency medical services (EMS) globally, and leadership challenges have been experienced worldwide regardless of the level of general healthcare or education [2]. According to previous research, the early phase of the pandemic was particularly difficult for EMS personnel, both mentally and physically. The leadership challenges during the pandemic have significantly impacted the well-being of EMS personnel, causing mental health problems, exceptional stress, and absences due to illness [3,4,5,6,7,8]. Previous research has identified several factors affecting well-being, with the main factors being difficulties in obtaining support or deficiencies in leadership. During the pandemic, paramedics felt left alone and without support from their superiors [4,5,6,8,9,10].

According to previous research, deficiencies in leadership were evident during the pandemic, including a lack of clear treatment protocols or constant changes, communication problems, and growing distance from supervisors [3,4,6,7,8,9,10]. In addition, in some countries with limited public healthcare, paramedics experienced a heavy workload, problems with equipment availability, and personal difficulties with mental health or burnout [3,4,10]. These results may have been influenced, for example, by a weaker healthcare capacity and higher COVID-19 mortality rates among patients or otherwise poor working conditions in EMS. This research takes place in Finland, a northern European country with approximately 5,550,000 inhabitants [11], where circa 774,000 prehospital EMS missions occurred in 2020 [12]. To ensure the security of healthcare resources in Finland, a state of emergency was declared, and the Emergency Powers Act was implemented on 17 March 2020 [13]. The purpose was to enable the implementation of additional measures and related recommendations and restrictions outlined by the government. For example, rules on rest periods and overtime in labor legislation and provisions of the annual leave law could be disregarded for healthcare personnel. Additionally, non-urgent healthcare services were restricted in the additional measures, and emergency healthcare, such as EMS, was strengthened [13].

Finnish organizations that provide prehospital EMS and their supervisors were in the midst of new challenges during the state of emergency. At the same time, the organizations had to care for patients who had become ill with a new and relatively unknown disease, run daily operations, and take care of employee well-being. The purpose of this study is to explore the paramedics’ experiences of work well-being related to leadership challenges during the early phase of the COVID-19 pandemic. The research question was: What kind of work well-being-related leadership challenges did paramedics experience during the early phase of the pandemic?

## 2. Materials and Methods

This qualitative study utilized reflective essay material written during the fall of 2020. The essays were gathered from a group of experienced advanced-level paramedics pursuing their master’s degree in the Development and Management of Emergency Medical Services at the South-Eastern Finland University of Applied Sciences. The essays were written as part of a course entitled “Current Issues in EMS Management”. A similar methodology has been utilized previously with other topics, yielding excellent reflective material [14,15].

The students were informed about the study, the privacy statement, and provided with the opportunity to ask further questions through a written research invitation on the Learn platform, published on 21 August 2020. The essay writing instructions were designed by the last author, a senior researcher focusing on EMS and work well-being topics, and the head of the master’s program in question. In the essays, the students were asked to describe, based on their own experiences and observations, how emergency conditions had affected the work well-being of EMS personnel. Three guiding questions were asked: (1) What kind of workload have/had EMS personnel experienced during the emergency conditions? (2) How has this workload differed from normal conditions? (3) What effects has/had this workload had on the well-being of the EMS personnel? The material contained a wealth of descriptions related to leadership and management and was thus suitable to approach from the perspective of the current study [16].

The essays were written during the period of 23 August 2020–30 November 2020. A total of 32 students out of 39 returned their essays during this time on the Moodle platform. All 32 students gave their informed consent to include their essays in the study. Only the essays written by students who participated actively in the EMS field work as advanced-level paramedics were included in the study (*n* = 30). The students worked in different regions across Finland, which were called hospital districts during the time of the study. None of the 20 hospital districts of Finland were over-represented. The final essay material was 106 pages long and contained a total of 32,621 words in Finnish.

### 2.1. Data Analysis

The material was analyzed following the inductive content analysis process [16,17,18]. First, the first author, a research-oriented, experienced, advanced-level paramedic, read the material multiple times to familiarize himself with the content. Words and sentences were chosen as a unit of analysis [16,17,18]. Then, the first author conducted the open coding phase and recorded headings and notes with content answering the research questions. The last author thoroughly reviewed the coding process. The coded material was grouped into subcategories, with similar content being combined. The created subcategories were named based on the content. Then, the subcategories were grouped into upper categories and named [16,17,18]. The categorization process was conducted in collaboration between the first and the last authors. The categorization was compiled into a figure, and the content of each upper category is elaborated in detail in the results section. Further, the results section provides quotes from the original material to substantiate the analysis conducted.

### 2.2. Ethical Considerations

Participation in the study was voluntary for the students. They had the option to decline, remove parts of their essays from the study material, or withdraw their essay completely at any time before the analysis began. No one used these options. They were made aware that their participation in the study would have no impact on the evaluation of their course. As stated in the research invitation, the last author removed the students’ names, ages, work experience years, and possible mentions of workplaces or locations before providing the research material to the first author.

Following the ethical principles of research with human participants and ethical review in the human sciences in Finland by the Finnish National Board on Research Integrity TENK, an ethical review statement from a human sciences ethics committee was not needed. The participants were adult students participating in normal work life, and participation in the research was based on informed consent. The physical integrity of the participants was not concerned, the participants were not exposed to exceptionally strong stimuli, the risk of causing mental harm did not exceed the limits of normal daily life, and participation did not involve safety threats [19]. South-Eastern Finland University of Applied Sciences approved the research request and gave permission for the study on 20 August 2020.

## 3. Results

The participating paramedics were 57% men. The majority of the participants were 30–39 years old, and they had several years of work experience in EMS (Table 1). The mean age of the participants was 34.9 years, and the mean work experience in the EMS was 8.7 years.

**Figure 1:**
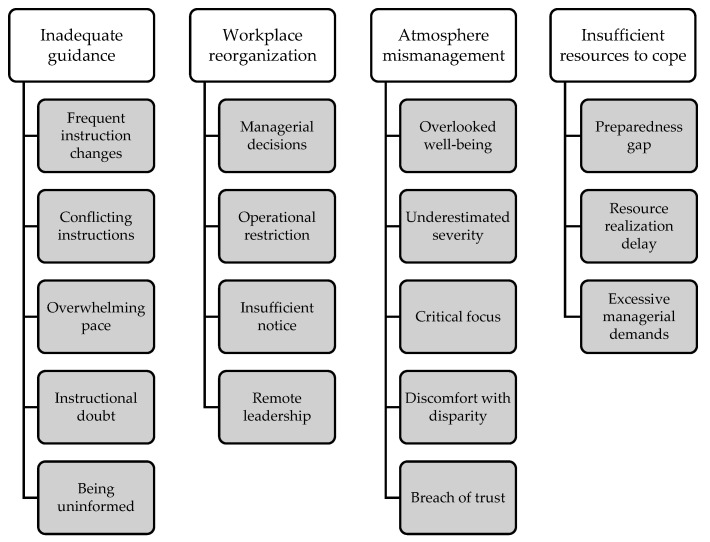
The upper categories and subcategories.

The work well-being-related leadership challenges that paramedics experienced during the early phase of the pandemic were divided into four upper categories: inadequate guidance, workplace reorganization, atmosphere mismanagement, and insufficient resources to cope (Figure 1). These upper categories were formed of a total of 17 subcategories.

### 3.1. Inadequate Guidance

**Frequent instruction changes:** Paramedics reported that a major challenge in the management during the early stages of the pandemic was the lack of and difficulties in guidance. Especially, the constant changes in guidelines and personal protective equipment recommendations were a burden on the personnel. The paramedics reported that the recommendations and guidelines frequently changed during the early stages of the pandemic, causing great uncertainty and frustration among the personnel.

*“The situation was not made easier by the constantly changing guidelines, which changed three times a day at best. It was also peculiar and confusing that there was no unified line of action and that the guidelines for the different emergency care providers in the region could vary and even change between shifts.”*

(Paramedic = P10)

**Conflicting instructions:** The multichannel nature of the guidance caused uncertainty among the paramedics. Guidance had come from multiple sources and through several different communication channels. The paramedics felt uncertain about what and whose instructions they should comply with.

**Overwhelming pace:** The paramedics also experienced difficulties internalizing and complying with the guidance. They felt that they did not have time to internalize the constantly changing instructions. The flood of information led to an experience of the futility of reading the instructions. They also became indifferent to the instructions, which was seen to impact work performance.

*“… the instructions changed too quickly, making it difficult to keep up. The constant flood of new and changing instructions had an additional impact on the workload. It is really difficult to keep track of the instructions and even have the motivation to be interested in them if the flood of information is overwhelming.”*

(P26)

**Instructional doubt:** The paramedics perceived the guidance quality as poor, leading to reduced confidence in the organization’s competence to provide proper instructions. This erosion of trust increased stress and reduced motivation among the personnel.

**Being uninformed:** Inadequate leadership left paramedics relying on uncertain information and insufficient and even incorrect guidance in the early stages of the pandemic. This sometimes led to incorrect action and significantly affected work well-being negatively.

### 3.2. Workplace Reorganization

**Managerial decisions:** During the early stages of the pandemic, the paramedics reported that, in many cases, supervisors were forced to make unilateral managerial orders, which put a strain on personnel. The employer unilaterally changed the personnel’s job description, and their established vacation times were changed during the pandemic to secure the needed employee resources.

*“This caused some employees to have changes in their work tasks. Some were asked about their willingness to change their work tasks, while others were not. The employer could only announce that these persons would be trained for the new task. Learning a new task is already mentally demanding, and having to switch to a new task against one’s own will surely hasn’t made things easier.”*

(P1)

**Operational restriction:** Additionally, work previously considered quite free was also restricted by other managerial orders regarding daily tasks, logistics, and interactions with others. This increased the personnel’s dissatisfaction.

**Insufficient notice:** The changes to the job description of the paramedics were quite sudden, and this affected their ability to perform their work.

*“I got the information on a [XX] at the end of March that I would start work on the next [XX] at 7:00… The warning time was four days!”*

(P17)

**Remote leadership:** During the early stages of the pandemic, the work of the paramedic supervisors was reorganized to be performed remotely. Paramedics felt that the transition of their supervisors to remote work created distance between the employees and supervisors. Consequently, there was a sense that the leadership turned impersonal and remote due to its reliance on virtual meetings and email communication.

### 3.3. Atmosphere Mismanagement

**Overlooked well-being:** The challenges of managing the work atmosphere were considered extremely difficult, as they were recognized to have long-term effects on the work community. Paramedics felt that their well-being was neglected in tough situations, with insufficient support and responsibility from their supervisors.

*“I believe that management should prioritize maintaining work well-being, but this has not been seen in my own workplace.”*

(P30)

**Underestimated severity:** Paramedics noted that during their most challenging situations, supervisors did not take the situation seriously. Some paramedics felt that the supervisors’ casual attitude had affected the experience of the severity of the situation.

**Critical focus:** Expressing a lack of support or leadership led to the supervisors questioning and criticizing the personnel’s feelings. In addition, the work atmosphere was hampered by the experience of criticism for even minor human errors.

*“When the lack of leadership was brought to the attention of the [XX], the paramedics received criticism, and it was directly expressed that the paramedics’ experience was wrong.”*

(P17)

**Discomfort with disparity:** The experience of working in conditions that exposed them to the risk of infection while their supervisors worked from a safer and more secure work environment caused resentment.

**Breach of trust:** The weakness of the work atmosphere and the failure to strengthen the atmosphere were seen in the community. The initial chaos and perceived absence of support for work endurance caused a loss of confidence in superiors and their ability to guide the personnel.

### 3.4. Insufficient Resources to Cope

**Preparedness gap:** Paramedics believed that the resources of organizations and supervisors for managing emergency conditions were insufficient. Paramedics felt that organizations were not prepared enough and, specifically, the adequacy of personnel during the pandemic had posed challenges.

*“In this first phase, colleagues who were involved described the atmosphere as downright chaotic and unbelievable because the situation came out of nowhere, and no one in our organization was prepared to lead such a crisis.”*

(P3)

**Resource realization delay:** According to the paramedics, the supervisors may not have fully understood the burden on the personnel quickly enough; partly for this reason, the personnel turnover had been exceptionally high during the pandemic.

**Excessive managerial demands:** Although paramedics criticized their supervisors’ behavior, they acknowledged that their supervisors were also at their limits during the pandemic’s early phase and were unlikely to have been able to alleviate the damaging situation of their work well-being.

*“With the supervisors also at their limits, it was understandable that they were not able to alleviate the reasons behind the negative attitudes of the employees.”*

(P14)

## 4. Discussion

The purpose of this study was to explore paramedics’ experiences in terms of work well-being related to leadership challenges during the early phase of the COVID-19 pandemic. The results were divided into four upper categories: inadequate guidance, workplace reorganization, atmosphere mismanagement, and insufficient resources to cope.

The challenges of directing personnel and particularly continuous communication, as well as constantly changing guidelines, were highlighted as a significant factor in reducing paramedics’ well-being during the pandemic’s early stages. International studies found similar results among paramedics [3,4,6,7,8,9,20] and extensive literature reviews among healthcare personnel in general [21,22]. The constant, excessive, sometimes poor-quality, multichannel communication was challenging to follow and caused frustration and uncertainty among paramedics in already unclear circumstances. These findings highlight the importance of adequate preparedness, which did not exist at the beginning of the pandemic [22]. Based on the results of the current study, paramedics also expressed an understanding that the situation had been challenging for decision makers and supervisors, who had likely felt obligated to disseminate the latest guidance to personnel as quickly as possible. The actions were well-intentioned, but the impact was not as intended as it further increased the frustration among paramedics due to information overload. Therefore, guidance and communication are clearly areas that require more attention [20] in similar situations in the future.

This study highlights challenges in work organization that were initiated by the employer, such as changes in personnel job descriptions or vacations. These changes were made with short notice during the pandemic and were permitted due to the state of emergency. Similar results were not found in international studies, which may indicate the diversity and multi-professionalism of Finnish EMS. Finnish advanced-level paramedics are also registered nurses with either a bachelor’s degree in prehospital emergency care (requiring 240 European Credit Transfer and Accumulation System (ECTS) from a university of applied sciences) or completion of a 30 ECTS advanced-level prehospital emergency care specialization course with a bachelor’s degree in nursing (210 ECTS) [23]. Therefore, paramedics can be utilized in a wider range of tasks. The changes to job descriptions and vacations were likely necessary from the organization’s perspective to ensure sufficient resources, and the experiences of challenges were therefore inevitable. However, anticipating these changes or informing personnel about them could be better performed in the future, making it an area that requires more attention. This is closely linked to inadequate guidance. These factors are also connected to effective crisis communication, whose principles and strategies [24] should be extendable to keeping personnel informed, aiming to foster a sense of security and belonging, activate employees as allies, and provide them with appreciation and support [25].

The distancing of leadership has been identified in international studies [6,8,9,10]. Similar to this study, paramedics felt that the visibility of leadership decreased as supervisors transitioned to remote work, and some paramedics felt that they were fighting against the pandemic without visible leadership. It should also be noted that, in Finland, remote work for supervisors was not their choice but was mandated by additional government measures [13]. However, this does not negate the need for visible leadership felt by paramedics, which should be a focus in the future. It is possible that some supervisors lacked the skills required for modern remote management. All organizations should invest in training for remote communication tools such as video conferencing software, which allows for face-to-face communication in remote team situations and improves visible leadership. Bringing up the faces of management regularly, even with the means of remote communication tools, has been seen to improve the experience of visible leadership in previous studies [9]. Additionally, employees who have adapted to receiving remote support and guidance during the pandemic will likely continue benefiting from these skills and operating models in normal circumstances.

According to a global study [2], the overall work environment in EMS during the pandemic was poor, and this study suggests that the situation was the same in Finland. Challenges in managing the work environment were evident, such as the lack of support provided by supervisors to personnel, downplaying the seriousness of the situation, or neglecting to take personnel into consideration. Similar experiences have led to declines in well-being in international studies as well [4,5,6,7,8,9,10]. Supervisors’ working hours during the pandemic were likely consumed by administrative challenges and ensuring sufficient personnel resources, leaving them with little time to support personnel. However, supporting and listening to personnel are among the top recommendations for promoting well-being in leadership [9,20]. According to an extensive systematic review, lack of support is connected to even turnover intention [26]. Therefore, support is a clear factor that supervisors should focus on in similar situations.

### Strengths and Limitations

The target group of this study consisted of Bachelor-level advanced-level paramedics who voluntarily applied to study for a Master’s degree. Most of them had extensive work experience in EMS and worked in the field during the early stages of the pandemic. This target group can be considered somewhat selective, aware, reflective, searching for connections and explanations, and willing to develop EMS. They are accustomed to writing reflective text due to their previously completed studies. The essays were chosen as material for this study since they could be considered reliable and high-quality descriptions of the matter based on the essay writers’ dispersion across Finland and their capabilities to describe their observations in depth because of advanced academic training. The students lived and worked in different parts of Finland, which obscured potential regional differences in experiences and, thus, it was indeed a strength that the study did not focus only on specific workplaces. In addition, 30 well-considered essays are an excellent quantity for qualitative research. For these reasons, the chosen target group is considered to have provided very rich data for this study, allowing for the exploration and understanding of the complexities and nuances of the phenomenon in question [27]. To increase credibility, the target group was described in detail [16].

The confirmability of the research [28] was ensured in several ways. The researchers did not have knowledge of previous research findings on the topic at the beginning of the analysis. They first conducted the data analysis and results reporting, then conducted a literature review to compile background literature on the topic. This allowed for them to approach the analysis with an open mind without being influenced by prior research. The first author who conducted the open coding phase deliberately avoided bringing his own experiences from EMS during the pandemic into the analysis [16] and proceeded with an inductive approach. The last author, who collected the data and participated closely in the analysis, was not directly involved in operational EMS work during the pandemic. The analysis was performed carefully in collaboration and by following the process of inductive content analysis [18], increasing the dependability. The results were presented transparently, and the credibility of the analysis was increased by showing direct quotes from multiple participants [16]. The process was described in detail to improve the research process’s confirmability and dependability further [29]. Finally, a thorough literature review confirming the findings increased the credibility [16].

This qualitative study did not aim for generalizability; instead, it provided rich and detailed descriptions of the participants’ experiences, adding insight and understanding of the topic. The transferability [26] of the paramedics’ experiences during the pandemic and the conclusions drawn to other emergency conditions cannot be estimated without additional studies. EMS is also only a small part of healthcare responsible for providing on-site emergency treatment in the case of sudden illness, injury, or worsening of a long-term illness. Still, the changes in guidelines and the use of multiple communication channels highlighted in this study have been present in other areas of healthcare [21,22,26], which somewhat increases the transferability of the study results.

## 5. Conclusions

Based on this study, the work well-being related to leadership challenges during the early phase of the COVID-19 pandemic from the viewpoint of paramedics included inadequate guidance, workplace reorganization, atmosphere mismanagement, and insufficient resources to cope.

Based on the challenges identified in leadership, one of the primary areas for improvement is clearly guiding personnel during emergency conditions. Mandatory changes to personnel will always cause discomfort but providing information as early and clearly as possible while explaining the importance of the changes is crucial. In addition, visible leadership posed challenges. Supervisors were forced to work remotely, and some undoubtedly lacked the necessary skills for modern remote management. To address this, every organization should prioritize investing in training programs for remote communication tools. Moreover, to improve the work atmosphere during emergency conditions, the focus should be on supporting personnel in various ways to maintain trust in the organization and collaboration under challenging situations.

Understanding the phenomena introduced in this study and implementing suggested actions might prevent the leadership challenges and the resulting decline in well-being in similar state-of-emergency situations. After the pandemic, there has been a severe global shortage of healthcare workers, including paramedics. The healthcare sector can no longer afford further decline in work well-being if we want to preserve the ability to handle similar state-of-emergency situations. Therefore, further studies should be aimed at determining the best practices for state-of-emergency leadership in healthcare. There is a dire need for studies to find the means to improve the attractiveness of the healthcare sector so as to ease the global shortage of healthcare workforce.

## Figures and Tables

**Table 1:** Background information of the participants (*n* = 30).

	*n*	%
**Gender**		
**Women**	13	43
**Men**	17	57
**Age**		
**<30 years**	7	23
**30–39 years**	17	57
**≥40 years**	6	20
**Work experience in EMS**		
**<6 years**	6	20
**6–9 years**	12	40
**≥10 years**	12	40

## Data Availability

The data presented in this study are available on request from the corresponding author. The data are not publicly available due to restrictions of the research permit and the confidentiality of the data communicated to the participants.

## Public Involvement Statement

Paramedics wrote the essays utilized in this study, their involvement in the study has been reported following the Reporting Patient and Public Involvement in Health and Social Care Research-checklist (https://www.equator-network.org/, accessed on 24 February 2023).

## Guidelines and Standards Statement

This manuscript was drafted against the Consolidated Criteria for Reporting Qualitative Research (https://www.equator-network.org/, accessed on 5 December 2023).
